# The mechanical and clinical influences of prosthetic index structure in Morse taper implant-abutment connection: a scoping review

**DOI:** 10.1186/s12903-023-03545-3

**Published:** 2023-10-21

**Authors:** Liheng Shen, Chengzhi Dong, Jianping Chen, Xiaolei Bai, Fan Yang, Linhong Wang

**Affiliations:** 1 Center for Plastic & Reconstructive Surgery, Department of Stomatology, Zhejiang Provincial People’s Hospital, Affiliated People’s Hospital, Hangzhou Medical College, Hangzhou, Zhejiang China; 2 Department of Stomatology, Zhejiang Chinese Medical University, Hangzhou, Zhejiang China; 3 Department of Stomatology, Zhejiang Provincial People’s Hospital, Affiliated People’s Hospital, Hangzhou Medical College, Hangzhou, Zhejiang China

**Keywords:** Implant-abutment connection, Morse taper, Prosthetic index, Review

## Abstract

**Aim:**

The implant-abutment connection is a crucial factor in determining the long-term stability of dental implants. The use of a prosthetic index structure in the Morse taper implant-abutment connection has been proposed as a potential solution to improve the accuracy of this connection. This study aimed to provide a scoping review of the mechanical and clinical effects of the prosthetic index structure in the Morse taper implant-abutment connection.

**Methods:**

A systematic scoping review of articles related to "dental implants," "Morse taper," and "index" was conducted using PubMed/MEDLINE, Web of Science, Cochrane, and Scopus databases, as well as a comprehensive literature search by two independent reviewers. Relevant articles were selected for analysis and discussion, with a specific focus on investigating the impact of prosthetic index structure on the mechanical and clinical aspects of Morse taper implant-abutment connections.

**Results:**

Finally, a total of 16 articles that met the inclusion criteria were included for data extraction and review. In vitro studies have demonstrated that the use of a prosthetic index structure in the Morse taper implant-abutment connection can affect stress distribution, biomechanical stability, and reverse torque values, which may reduce stress within cancellous bone and help limit crestal bone resorption. However, retrospective clinical studies have shown that this structure is also associated with a higher risk of mechanical complications, such as abutment fracture and abutment screw loosening.

**Conclusions:**

Therefore, the clinical trade-off between preventing crestal bone resorption and mechanical complications must be carefully considered when selecting appropriate abutments. The findings suggest that this structure can improve the accuracy and stability of the implant-abutment connection, but its use should be carefully evaluated in clinical practice.

**Supplementary Information:**

The online version contains supplementary material available at 10.1186/s12903-023-03545-3.

## Introduction

Dental implants are widely used in the treatment of partially or totally edentulous patients. The success of the prostheses along with bone level stability and soft tissue health maintenance around dental implants are key factors for long-term success of implant therapy [1]. The implant-abutment connections are the weakest points of the implant, which could affect marginal bone loss [2]. Although there are a large number of implant designs on the market, the implant-abutment connection design can be divided into two main categories: external connection and internal connection [3]. The internal connections can be further divided into internal hex (hexagons, octagons), internal conical and Morse taper connections [4]. Various studies proposed that the internal conical connections could have generated better results in terms of abutment fit, stability, distribution of the functional loads, better seal performance, and less marginal bone loss [2, 5–7]. In particular, the Morse taper connection is widely recognized for its superior performance in terms of implant survival, success, and marginal bone loss [4, 6, 8].

Currently, various Morse-taper connection implants have been developed, offering a wide range of abutment options [9]. Most of these abutments consist of two parts, namely an abutment and a passing screw. The two-piece design incorporates an index, similar to an internal hexagon connection, to ensure proper alignment with the implant [10]. Recent studies have indicated that the presence of the index on Morse-taper abutments can modify or prevent the frictional effect typically observed with solid one-piece abutments [11–13].

Conversely, the introduction of indexed Morse-taper abutments has brought about certain advantages and simplifications in implant-supported rehabilitation procedures. It is more difficult to ensure the accuracy of the Morse taper (MT) abutment position because the non-indexed (NI) Morse taper abutment is a smooth internal surface and lacks installation guidelines [11]. In addition, inaccurate positioning between the implant and abutment may lead to microleakage [14] and affect the long-term stability of the oral restoration [15]. Furthermore, it has addressed some of the challenges encountered with conventional Morse-taper connections lacking an index. However, abutments without index could be assembled to the implants with index. In these conditions, the higher empty space between implant and abutment could facilitate the microleakage and bacterial colonization [16]. The microspace created by the microgap between the implant and abutment facilitates the penetration of macromolecules in tissue fluids and saliva, promoting bacterial invasion and proliferation, which eventually leads to bone loss, and this leakage is a major contributing factor for peri-implantitis [17]. To improve abutment positioning on Morse taper implants, manufacturers have added an internal positioning indicator and developed implants with indexed MT restorative connections that add an anti-rotation system similar to an internal hex to the Morse taper connection, and they indicated that the added internal octagon indexing did not significantly reduce the strength of the Morse taper implant connection [10, 18].

Undeniably, the addition of the internal hexagonal structure allows for a more precise connection between the Morse taper abutment and the implant. According to the manufacturer’s instructions, a Morse taper abutment containing a hexagonal structure requires a proper screw as well as a low mounting torque, which raises concerns about the final torque and life of this abutment. Villarinho et al. reported that the presence of an index may negatively affect the biomechanical stability of cementation screws in tapered abutments [11]. The potential for implant abutment fracture (AF) increases when implant loading is stressed, but managing abutment fracture in a clinical setting remains challenging. However, questions persist regarding whether the incorporation of this prosthetic index structure has an impact on the mechanical strength of the connection between the Morse taper abutment and the implant, its overall stability, and its potential to induce microleakage. Furthermore, the clinical ramifications of Morse-tapered abutments featuring a prosthetic index remain uncertain. The research questions guiding this study are as follows: "What is the comprehensive impact of prosthetic index structures on Morse taper implant-abutment connections, considering both their mechanical and clinical influences?".

## Methods

### Protocol

The protocol of this scoping review was developed in PROSPERO (www.crd.york.ac.uk/prospero). This review was performed following the Cochrane Handbook for Scoping review. The PRISMA statement were followed by all of the authors to report the results [19]. Lh Shen and Cz Dong were tasked with the acquisition of data, and they independently conducted the analysis and interpretation of the data. Lh Wang and F Yang played pivotal roles in conceiving and designing the study. Furthermore, inter-examiner agreement tests were conducted to validate the reliability and consistency of our data.

Using the population-context-concept (PCC) framework helps us to systematically identify and categorize relevant studies and information for scoping reviews, which allows us to clarify the population of interest, the context in which the study was conducted, and the core concepts we aim to explore [20]. Here, we clarify the PCC framework for this study as follows:

1. Population: Patients with Morse taper implant-abutment connections and model in vitro involved in Morse taper connections.
2. Context: Dental clinics, hospitals, and laboratory.
3. Concept: Mechanical and clinical influences of prosthetic index structure in Morse taper implant-abutment connections.

### Eligibility criteria

#### Inclusion criteria

1. Type of study: clinical or in vitro studies involving the Morse taper implant-abutment connection.
2. Intervention/exposure factors: studies investigating the use of restorative index structures in the Morse taper implant-abutment connection. Studies investigating various types or designs of restorative index structures in Morse taper connection.
3. Outcomes: studies reporting in vitro experimental outcomes (e.g., stability, torsional resistance, microleakage, etc.) related to the Morse taper implant-abutment connection and restorative index structures, or clinical outcomes (e.g., implant success, survival, abutment complications, etc.) related to restorative index structures in the Morse taper connection.

#### Exclusion criteria

1. Studies not related to the mechanical or clinical effects of the restorative index structure in the Morse taper implant-abutment connection.
2. Studies with insufficient data or unclear methodology.
3. Review articles, opinion pieces and conference abstracts.

### Information sources, search strategy, and study selection

A comprehensive search was conducted on PubMed/MEDLINE, Web of Science, Cochrane, and Scopus databases until February 2023 to identify in vitro and in vivo studies addressing the influence of prosthetic index structure in Morse taper implant-abutment connection. The search keywords included (dental implants OR dental abutment) AND (index OR hex) AND (internal connection OR conical connection OR Morse Taper) (searching strategies are supported in Additional file 1). The full texts or summaries of all studies, reports, and conference abstracts resulting from the advanced search were extracted. Following a thorough screening of titles, abstracts, and full texts, we removed duplicate entries to exclude unrelated studies and select relevant ones. The articles considered for this study encompassed randomized controlled trials, non-randomized studies evaluating intervention effects, and systematic reviews (the PRISMA flow diagram is presented in Additional file 2) [19].

## Results

**Fig. 1:**
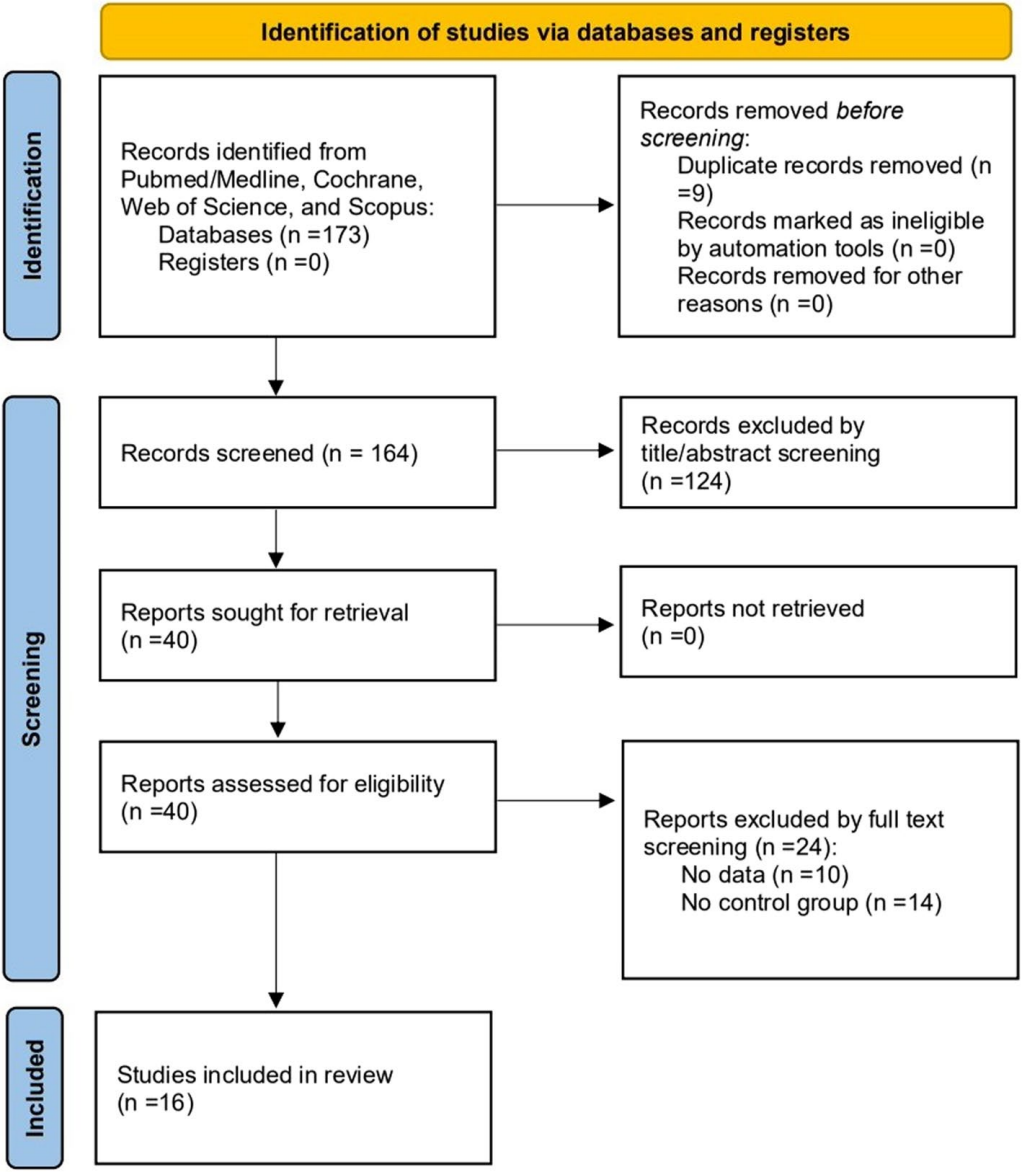
Flow chart of articles selection

According to PCC in this scoping review, title and abstract reviews were performed to identify the articles that met the review objectives. The search generated 173 titles initially. Out of these, 9 articles were screened after the removal of duplicates. About 124 articles were further excluded as they did not meet the eligibility criteria. This left us with 40 articles, out of which 24 articles were further excluded after full-text screening. Finally, 16 articles were included in the final review (Fig. 1), which were divided into two categories, in vitro experiment and clinical manifestations, in which the in vitro experiment was divided into bacterial seal performance and mechanical performance.

### Index structure design

The Morse taper internal connection is an implant-abutment connection, which is used to attach the abutment or other repair component to the implant. The Morse taper connection was first introduced in dental implants by the Swiss company, Straumann, in the mid-1990s [21]. The Morse taper connection has since become a popular connection system used by many dental implant manufacturers due to its reliable and stable connection, which allows for high implant stability and reduced micromovement [6]. The Morse taper of the abutment varies from manufacturer to manufacturer and basically ranges from 1 to 12 degrees. In recent years, some manufacturers have added a prosthetic index to the connection system, aiming at a more precise connection and better resistance to rotation [10, 18].

**Fig. 2:**
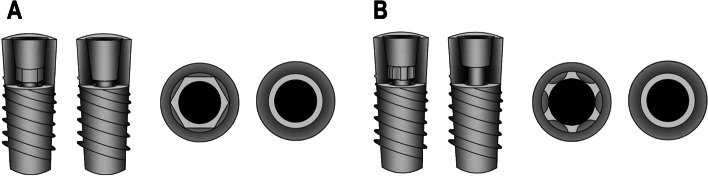
Comparison of coronal and horizontal planes between a common Morse taper abutment and a Morse taper abutment containing a hexagonal indexing repair component: **A** Without changing the height of the abutment, the lower part of the Morse taper abutment is changed to the inner hexagonal structure. **B** The height of the abutment is increased, and an additional internal hexagonal structure is added below the abutment

The geometric design of the prosthetic Index also differs, the common clinical ones are internal hexagonal (Nobel Active, Nobel Biocare AB, Göteborg, Sweden), internal octagonal (Cowell Medi, Busan, South Korea), implant–- abutment connection with six cams and grooves (Ankylos C/X, FRIADENT GmbH, Mannheim, Germany), and cams and grooves (Bone Level, Institut Straumann AG, Basel, Switzerland), etc. [22]. Some abutments (such as Ankylos C/X, FRIADENT GmbH, Mannheim, Germany) have a reduced surface area at Morse taper joints due to the addition of a prosthetic index (Fig. 2A), while others have a prosthetic index, while other abutments increased the prosthetic index from the original Morse taper and showed no change in the surface area of the Morse taper (Fig. 2B).

The prosthetic index of abutment could guide the prosthetic component into the implant. However, compared to abutments without an index, the mechanical properties and clinical impact of the prosthetic indexes within the Morse taper abutment on the abutment remains unclear. In 2009, Semper et al. evaluated different implant positional index design including regular polygonal, polygon profile, and cam-groove patterns from the theoretical considerations, they found that the rotational freedom of positional indices can be analytically calculated and is influenced by different parameters. The rotational freedom of the positional index of commonly used implant-abutment connections depends on their geometric design and size [23].

### In vitro studies

#### Bacterial seal performance

Unstable implant-abutment structures can lead to microleakage at the implant-abutment interface, followed by bacterial contamination leading to crestal bone loss or peri-implantitis [17, 24, 25]. Optimal adaptation, minimal micromotion, high-precision restorations, and an ideal occlusal relationship are the factors that can prevent or reduce microleakage. The tapered interface of the Morse Taper implant has a high contact area, which reduces the gap and helps to achieve an effective seal between the implant and the abutment. This high-precision contact preempts relative movement between the two, reducing screw tightening, microleakage, peri-implant inflammation, and preserving peri-implant bone. The external-hexagon implant configuration showed the greatest microleakage, followed by internal-trilobe, internal-hexagon, and internal-taper configurations [26].

Most of the studies focused on the differences in microleakage between internal and external connections, inner conical and hexagon. Two of the sixteen studies we included performed in vitro experiments to compare the effect of the presence or absence of an index on microleakage in internal cones (Table 1). Resende et al. evaluated the presence of prosthetic index assembled to the Morse taper implants by bacterial microleakage test in static conditions, they found that the presence of the internal index of the restoration does not affect the bacterial microleakage of the Morse cone implant [16]. Peruzetto et al. compared the bacterial seal at the implant abutment interface using two Morse taper implant models by in vitro microbiological analysis, they found that both tapered components failed to provide adequate sealing to bacterial leakage, although the indexed type components showed a superior seal compared with non-indexed components [9].

**Table 1:** In vitro study of the restorative index on microleakage of the implant-abutment connection

Reference	Method	Specie of bacteria	Results or Implications
Resende et al., 2015 [16]	In vitro co-culture of Implant-Abutment Complex and bacteria	*Streptococcus sanguinis*	The seal provided by the Morse Tapered Implant-Abutment Complex is not sufficient to protect the implant from bacterial penetration, whether or not it contains a prosthetic index
Peruzetto et al., 2016 [9]	In vitro co-culture of Implant-Abutment Complex and bacteria	*Escherichia coli*	Index abutments show better sealing than non-index abutments, but still suffer from bacterial microleakage

### Mechanical performance

The mechanical properties of abutments are a major concern for dentists, scholars, and implant manufacturers. Twelve of the sixteen papers we included on Morse taper abutments for prosthetic indexes were studies on the mechanical properties of this construction on abutments. The most frequent mechanical complications are loss of preload, abutment screw loosening (ASL), abutment or implant fracture, and deformations at the different interfaces [3]. The passivity, adaptation, and connection strength of the abutment to the implant are essential requirements for successful implant treatment [27]. The external hexagon was the first anti-rotation system in implant dentistry, after which Morse cone implants with indexed prosthetic connections were developed for more precise positioning between the abutment and the implant. The influence of the restorative index on the restoration of the Morse taper abutment on the implant stability has been evaluated using three-dimensional finite element analysis (FEA) [11, 28–30] (Table 2). The FEA of Zancopé et al. showed that the prosthetic index zone was in an out-of-stress condition and that the presence of the prosthetic index on the Morse tapered implant did not reduce the fracture resistance of the implant [28]. Zhang et al. investigated the stress distribution of three implant-abutment connections using three-dimensional finite element analysis: the Morse taper connection with platform switching (MT-PS) implant system with or without index and the internal hex connection with platform matching (IH-PM) implant system. Under vertical and inclined loading, the MT-PS implant system had significantly higher stress levels in the abutment neck and lower stress levels around the peri-implant bone compared to the IH-PM implant system. It concluded that MT-PS with indexing could reduce stresses within cancellous bone, but lead to higher stress concentrations in the abutment neck and greater susceptibility to mechanical complications [29]. Villarinh's study also supports that indexed tapered abutments may induce greater biomechanical risk in single-crown restorations when under function. The nonindexed group exhibited a higher initial preload compared to the indexed group. After mechanical cycling, the preload decreased in both the nonindexed and indexed groups, with a smaller reduction in the non-indexed group [11]. In vitro*,* loading of a Morse taper implant system containing an internal hexagon was found to have severe deformation at the interface between the internal hexagon and the Morse taper intersection. The internal hexagonal connection has higher compressive strength than the internal hexagonal connection combined with Morse taper design [30]. The incorporation of the internal hexagonal connection into the Morse taper design reduces the rotation of the abutment, however, it decreases the compressive strength of the Morse taper abutment. The presence of a prosthetic index on Morse taper abutments did not influence the resistance to fracture [31].

**Table 2:** In vitro studies on the mechanical properties of the implant-abutment connection by the prosthetic index

Reference	Method	Model	Implant and/or abutment	Results or Implications
Zancope et al., 2017 [28]	Bending Test and Fatigue Loading Test	Three-dimensional finite element model	Morse taper implants, with 11.5-degree angulation of the internal conical portion	The presence of the prosthetic index on the Morse tapered implant did not reduce the fracture resistance of the implant
Zhang et al., 2022 [29]	Loading Test	Three-dimensional finite element model	3.5 mm × 11 mm bone level implants with an indexed component, Ankylos, Dentsply Friadent GmbH, Mannheim, Germany	Morse taper connection with platform switching (MT-PS) with indexing could reduce stresses within cancellous bone, but lead to higher stress concentrations in the abutment neck and greater susceptibility to mechanical complications
Perriard et al., 2002 [18]	Loading test and fatigue tests	Resin block model and three-dimensional finite element model	standard 6 degrees taper 7 mm height abutment (Straumann, Waldenburg, Switzerland)	The mechanical resistance of the abutment to bending and torsional forces is equal regardless of whether the prosthetic index is present
Hung et al., 2019 [30]	Torque/detorque test and loading test	Three-dimensional finite element model	5 mm × 11 mm implants with an indexed component, Shinhung MST	Internal hex connection has higher compressive strength than internal hex connection combined with Morse taper design
Villarinho et al., 2017 [11]	Mechanical cycling and Removal torque and tensile removal force tests	Resin block model	3.75 mm × 11 mm Morse taper connection implants (Neodent, Curitiba, Brazil)	Indexed tapered abutments may induce greater biomechanical risk in single-crown restorations when under function
Cerutti-Kopplin et al., 2014 [32]	Reverse torque tests	Stainless-steel block model	4.3 mm × 10 mm implants with Morse taper connections (Alvim CM; Neodent) with an internal hexagona	There was no significant difference in reverse torque between indexed and non-indexed support groups
Martins et al., 2019 [13]	Mechanical fatigue test	Resin block model	3.5 mm × 11.5 mm implants with prosthetic Morse taper connection (Pross Implantes, RibeirãoPreto, SP, Brazil)	Indexed abutment retaining screws were more susceptible to loosening during reverse torque testing than non-indexed abutments
Hyun et al., 2020 [33]	Compressive strength tests	Resin block model	4 mm bone level implants with an indexed component (ComMed, Chang Gung medical technology Co., Ltd, Taiwan)	The increase in contact area resulted in less variation in rotation of Morse tapered abutments containing the positioning index
Yao et al., 2015 [22]	Antirotational tests	Resin block model	5.0 × 10 mm implant with a conical connection (taper angle = 7°) (CowellMedi, Busan, South Korea)	Adding a positioning (octagonal) index to the taper connection provides resistance to rotation but affects the flexural strength of the abutment
De Oliveira et al., 2016 [12]	Removal torque and tensile removal force test after thermomechanical cycling	Resin block model	4 mm × 13 mm implants with an indexed component (Titamax CM Cortical, Instradent AG)	Morse tapered abutments with positioning (hexagon) index have good resistance to rotation
Michelon et al., 2019 [34]	Mechanical cycling and postcycling tensile strength test	Resin block model	Morse taper connection implants, 3.5 × 11.0 mm with 11.5-degree internal angle conical connection	Cyclic loading increased the tensile strength of the Morse tapered implants regardless of the presence of an index. The presence of the index did not significantly change the tensile test values before and after cyclic loading
Nokar et al., 2020 [35]	Torque/detorque test and loading test	Stainless-steel block model	4.5 mm × 10 mm bone level implants (Implantium, Dentium Co, Seoul, South Korea)	Abutments with indexes do not have reduced flexural capacity under static loading compared to abutments without prosthetic indexes

To facilitate the restorative procedure, an index structure such as an internal hexagon or internal octagon was introduced into the Morse taper system, which might lead to changes in the stability of the implant-abutment complex. Cerutti-Kopplin et al. found no significant difference in reverse torque between the indexed abutment-containing group and the non-indexed group [32]. However, an experimental study demonstrated that indexed abutment retaining screws were more susceptible to loosening during reverse torque testing than non-indexed abutments [13]. Hyun et al. used FEA to find that there was no significant difference in the contact area between abutments of different shapes and implants, but the positioning hexagonal design of the Morse-tapered abutment acted against the rotational torque. The hexagonal shape of the abutment and the corresponding hexagonal contact area resulted in less rotational variation when the rotational moment was applied to a Morse-tapered abutment containing a positioning hexagon [33]. After the incorporation of the internal hexagonal index in the Morse taper abutment, the reduction of the tapered area of the abutment may lead to a biomechanical disadvantage of the Morse taper connection. Yao et al. demonstrated that in the Cowell implant system (taper angle = 7°), a purely tapered connection has no resistance to rotation. Adding an octagonal index provides resistance to rotation, but affects the flexural strength of the abutment [22]. Otherwise, it has been shown that abutment type has no significant effect on the removal torque and tensile removal force after mechanical cycling. The hexagonal shape of the abutment and the corresponding hexagonal shape inside the implant have corresponding contact surfaces. When the rotational moment is applied to the Morse-tapered abutment containing the positioning hexagon, the rotational variation is small [12].

### Clinical performance

How the implant and abutment are attached has long been considered to be closely related to the long-term prognosis of the implant. This paper focuses on the effect of the presence or absence of a positioning index on implants containing a Morse taper. However, there are fewer relevant clinical studies. Most studies have focused on the comparison between Morse taper and internal hex. In a meta-analysis that included 14 randomized clinical trials or prospective studies, the kind of implant-abutment connection had an impact on peri-implant bone loss. And when an internal interface was used, alveolar ridge bone levels were better maintained in the short to medium term, however, the Morse taper connection appeared to be more favorable, showing lower peri-implant bone loss [2]. A retrospective clinical study by Szyszkowski et al. with 540 implants showed a significantly lower mean marginal bone loss in the internal cone compared to the internal hex group [38].

Although there is a large body of literature on Morse taper in internal connections. Of the sixteen papers we included, there were only two relevant clinical studies on Morse taper abutments containing a prosthetic index [36, 37] (Table 3). Yang et al. followed 945 implants from 495 patients for one to nine years and found that the Morse cone connection is a safe abutment connection. This is one of the few retrospective clinical studies involving abutment localization indices with Morse taper. It was found that abutment fracture (AF) often occurs in abutments containing a positioning index (/X) [36]. More clinical studies are needed to produce more valuable evidence-based evidence to support good long-term clinical outcomes. Gehrke et al. evaluate the behavior of Morse-taper indexed abutments by analyzing the marginal bone level (MBL) after at least 12 months of function by a retrospective clinical study [37]. The average MBL was − 0.67 ± 0.65 mm in mesial and − 0.70 ± 0.63 mm in distal (*p* = 0.5072). The most important finding was the statistically significant difference comparing the values obtained for MBL between the abutments with different transmucosal height portions, which were better for abutments with heights greater than 2.5 mm. Regarding the diameter of the abutments, 58 had 3.5 mm (53.2%) and 51 had 4.5 mm (46.8%). There was no statistical difference between them, with the following means and standard deviation, respectively, − 0.57 ± 0.53 mm (mesial) and − 0.66 ± 0.50 mm (distal), and − 0.78 ± 0.75 mm (mesial) and − 0.746 ± 0.76 mm (distal). Regarding the implant dimensions, 24 implants were 3.5 mm (22%), and 85 implants (78%) had 4.0 mm. In length, 51 implants had 9 mm (46.8%), 25 had 11 mm (22.9%), and 33 implants were 13 mm (30.3%). There was no statistical difference between the abutment diameters (p > 0.05). The above-mentioned study concluded that better behavior and lesser marginal bone loss were observed when using abutment heights greater than 2.5 mm of transmucosal portion and when placed implants with 13 mm length. Furthermore, this type of abutment showed a little incidence of failures within the period analyzed in the study [37].

**Table 3:** In vivo study of the clinical performance of the prosthetic index on the implant-abutment connection

Reference	Research type	Number of Implant	Years	Results or Implications
Yang et al., 2022 [36]	Retrospective clinical study	945 implants	One to nine years	Abutment fracture (AF) often occurs in abutments containing a positioning index
Gehrke et al., 2023 [37]	Retrospective clinical study	109 implants	At least > 1 year, average 22.7 months	Marginal bone loss is less when using an abutment height greater than 2.5 mm transmucosal portion and when placing implants with a length of 13 mm. The indexed Morse tapered abutment had no mechanical complications during the follow-up period

## Discussion

Different implant-abutment connections, such as internal hexagonal connections and Morse taper connections, have been developed to mitigate biological complications like marginal bone loss (MBL) and mechanical complications including abutment fracture. These alternative connections facilitate enhanced transmission of occlusal loads to the bone and implant, while establishing a more secure interface between the abutment and implant, thereby reducing microgaps and the subsequent risk of bacterial colonization. Although implant-abutment complexes attached by tapered connections also have problems related to bacterial colonization, some studies have confirmed that tapered connections do have better post-5-year clinical outcomes. In a meta-analysis incorporating 14 randomized clinical trials and prospective studies, the type of implant-abutment connection was found to influence peri-implant bone loss. Employment of an internal interface exhibited superior maintenance of alveolar ridge bone levels in the short-to-medium term; however, the Morse taper connection appeared more advantageous, exhibiting reduced peri-implant bone loss [2]. Furthermore, a retrospective clinical study conducted by Szyszkowski et al., involving 540 implants, demonstrated significantly lower average marginal bone loss in the internal cone group compared to the internal hex group [38].

Following the conventional procedure, a seal is created by properly twisting the central retaining screw of the abutment while securing the Morse taper abutment. However, upon closer inspection, a mismatch between the surfaces of the Morse taper abutment and the implant connection may be found and micro-movement wear may occur in the future. Studies have shown that re-fixing the central screw will result in a reduction in the reverse torque value of the abutment. Once the implant begins to occlude the load, microfractures form internally between the implant and the abutment. After bacterial colonization and multiplication, peri-implant mucositis and peri-implantitis can eventually develop, leading to marginal bone resorption (MBL) and even implant failure.

In recent years, to achieve more accurate positioning and seating of the Morse taper abutment, developers have investigated modified Morse taper abutments by changing to prosthetic indexes in the lower part of the Morse taper (Fig. 2A) or by adding additional prosthetic indexes at the lower end (Fig. 2B). Such internal hexagonal or octagonal indexes can be provided to components with tapered connections for more accurate positioning, thereby facilitating restorative procedures. This construction allows for a tighter and more stable connection between the abutment and the implant, thus reducing bacterial microleakage, but may affect the mechanical properties and clinical performance of the Morse tapered abutment. Current research in this area relies on three-dimensional finite element analysis, bacterial leakage experiments, and a limited number of long-term retrospective clinical observational studies. Therefore, the purpose of this study was to provide a little guidance to clinicians when using Morse taper abutments with prosthetic indexes by examining existing in vitro studies (Tables 1 and 2) and clinical trials (Table 3). Peruzetto et al. compared the bacterial seal at the implant abutment interface using two Morse taper implant models by in vitro microbiological analysis, they found that both tapered components failed to provide adequate sealing to bacterial leakage, although the indexed type components showed a superior seal compared with non-indexed components [9]. Furthermore, another study found no significant difference in bacterial microleakage between the utilization of a prosthetic index and non-index abutment under static conditions [16]. To date, reports on this topic are currently limited, and there is no unified conclusion on this issue.

In vitro experiments confirmed that the positioning (hexagonal) index of Morse tapered abutments has good resistance to rotation [12, 33]. While prosthetic indexes could improve the accuracy of restorative procedures and increase their resistance to rotation, it may reduce the internal tapered area of contact with the abutment (Fig. 2A), which could potentially affect mechanical stability. Yao et al. confirmed that the positioning (hexagonal) index of Morse tapered abutments has good resistance to rotation, but reduced resistance to bending [22]. Another in vitro study investigated the effect of a positioning index on the abutment screw preload values of tapered connection implants. It might be concluded that the presence of a positioning index might negatively affect the biomechanical stability of the tapered abutment screws and therefore their long-term prognosis when applied to single implant-supported cemented restorations [11]. Hung et al. confirmed the reduced compression resistance of Morse taper abutments with prosthetic indexes compared to internal hexagonal connections [30], and Martin et al. concluded that indexed Morse taper abutments are more prone to abutment screw loosening after reverse torque loading [13]. A previous study evaluated the effects of the prosthetic index on the stress distribution in Morse taper connection implant systems and peri-implant bone. The use of three-dimensional finite element analysis by Zhang et al. is a noteworthy advantage because of its ability to accurately display and measure the stress distribution within the implant system and peri-implant bone [29]. These findings indicated that the Morse taper connection with platform switching (MT-PS) with the index will cause higher stress concentration on the abutment neck than those without index, which is more prone to mechanical complications. MT-PS decreases stress within the cancellous bone and may help limit crestal bone resorption. However, this study also has certain limitations, including the assumption of idealized conditions in the simulations, the failure to take into account the complex mechanical and biological interactions that occur in vivo, and the failure to take into account other factors that may affect the stress distribution, such as implant diameter, length, and thread design. It is worth noting that a variety of other factors may be involved in obtaining different results [29]. However, a separate study indicated that that the presence of a prosthetic index did not decrease the implants' resistance to fracture [28]. In addition, some in vitro studies have pointed out that the resistance to bending, torsion, and tension of the Morse taper abutments is not significantly different with or without the prosthetic index [18, 32, 34, 35].

Limited research has been conducted on clinical studies, and to date, only two retrospective studies have been identified as relevant to this aspect (Table 3). A retrospective study of 1 to 9 years evaluated the cumulative mechanical complications of the Morse taper connection with and without a prosthetic index from a biomechanical perspective over 1 to 9 years. A total of 25 cumulative abutment mechanical complications (2.65%) occurred in 945 implants, including AF (*n* = 13, 1.38%) and ASL (*n* = 12, 1.27%). The study also showed that the presence of an index may impair mechanical performance and that abutments with a prosthetic index had a higher incidence of AF than those without an index [36]. These findings highlighted the importance of carefully selecting the appropriate prosthetic index to ensure optimal clinical outcomes and reduce the risk of abutment fracture in clinical practice, taking both biomechanics and clinical research perspectives into account. Gehrke et al. evaluate the clinical performance of indexed Morse-taper abutments by analyzing the marginal bone level after at least 12 months of function in 109 implants by a retrospective clinical study [37]. The above-mentioned study concluded that better behavior and lesser marginal bone loss were observed when using abutment heights greater than 2.5 mm of transmucosal portion and when placed implants with 13 mm length. Furthermore, this type of abutment showed a little incidence of failures within the period analyzed in the study. A larger sample size of clinical randomized controlled trials is needed to further analyze the effect of a Morse taper abutment with prosthetic index on long-term clinical outcomes.

## Limitations

One of the limitations of this study was the inherent heterogeneity among the various studies, including the diversity in study designs, methodologies and so on. Additionally, the shape and design of prosthetic indexes varied considerably from one implant system to another. This variability posed difficulties when attempting to establish universal comparisons or generalizations across different implant systems. Furthermore, in the realm of clinical research, our analysis primarily relied on retrospective studies due to the availability of relevant data. These retrospective studies had limitations, such as relatively small total sample sizes and low incidence rates of specific outcomes. These factors, in turn, introduced potential biases and limitations to our review.

## Conclusion

Within the limitation of this review, the incorporation of the prosthetic index into the Morse tapered abutment offers advantages in terms of rotational resistance, However, this benefit comes with a trade-off, as it reduces the fracture resistance of the abutment. Our comprehensive analysis of both in vitro experimental studies and in vivo clinical retrospective studies consistently indicates that indexed abutments introduce stress concentrations in the abutment neck, rendering them more susceptible to mechanical complications. Therefore, clinicians should take the prosthetic index into account when selecting appropriate abutments. Further research is needed to validate these findings and explore the implications of abutment structures on stress distribution in implant systems and long-term success.

### Supplementary Information

**Additional file 1.**

**Additional file 2.**

## Data Availability

The datasets used and/or analysed during the current study available from the corresponding author on reasonable request.

## Abbreviations

MT: Morse taper
IN: Indexed
NI: Non-indexed
MT-PS: Morse taper connection with platform switching
MBL: Marginal bone level
FEA: Finite element analysis
AF: Abutment fracture
ASL: Abutment screw loosening

## References

[1] Thoma DS, Mühlemann S, Jung RE (2014). Critical soft-tissue dimensions with dental implants and treatment concepts. Periodontol 2000.

[2] Caricasulo R, Malchiodi L, Ghensi P, Fantozzi G, Cucchi A (2018). The influence of implant-abutment connection to peri-implant bone loss: A systematic review and meta-analysis. Clin Implant Dent Relat Res.

[3] Vinhas AS, Aroso C, Salazar F, López-Jarana P, Ríos-Santos JV, Herrero-Climent M (2020). Review of the mechanical behavior of different implant-abutment connections. Int J Environ Res Public Health.

[4] Goiato MC, Pellizzer EP, da Silva EV, Bonatto Lda R, dos Santos DM (2015). Is the internal connection more efficient than external connection in mechanical, biological, and esthetical point of views? A systematic review. Oral Maxillofac Surg.

[5] Galindo-Moreno P, Concha-Jeronimo A, Lopez-Chaichio L, Rodriguez-Alvarez R, Sanchez-Fernandez E, Padial-Molina M. Marginal Bone Loss around Implants with Internal Hexagonal and Internal Conical Connections: A 12-Month Randomized Pilot Study. J Clin Med. 2021;10(22):5427. 10.3390/jcm10225427 10.3390/jcm10225427 PMC8621760 34830709

[6] Schmitt CM, Nogueira-Filho G, Tenenbaum HC (2014). Performance of conical abutment (Morse Taper) connection implants: a systematic review. J Biomed Mater Res A.

[7] Larrucea Verdugo C, Jaramillo Núñez G, Acevedo Avila A, Larrucea San Martín C. Microleakage of the prosthetic abutment/implant interface with internal and external connection: in vitro study. Clin Oral Implants Res. 2014;25(9):1078–83. 10.1111/clr.12217. 10.1111/clr.12217 23822097

[8] Vetromilla BM, Brondani LP, Pereira-Cenci T, Bergoli CD (2019). Influence of different implant-abutment connection designs on the mechanical and biological behavior of single-tooth implants in the maxillary esthetic zone: A systematic review. J Prosthet Dent.

[9] Peruzetto WM, Martinez EF, Peruzzo DC, Joly JC, Napimoga MH (2016). Microbiological seal of two types of tapered implant connections. Braz Dent J.

[10] Ding TA, Woody RD, Higginbottom FL, Miller BH (2003). Evaluation of the ITI Morse taper implant/abutment design with an internal modification. Int J Oral Maxillofac Implants.

[11] Villarinho EA, Cervieri A, Shinkai RS, Grossi ML, Teixeira ER (2015). The effect of a positioning index on the biomechanical stability of tapered implant-abutment connections. J Oral Implantol.

[12] de Oliveira Silva TS, Mendes Alencar SM, da Silva VV, de Moura CDVS (2017). Effect of internal hexagonal index on removal torque and tensile removal force of different Morse taper connection abutments. J Prosthet Dent.

[13] Martins CM, Ramos EV, Kreve S (2019). Reverse torque evaluation in indexed and nonindexed abutments of Morse Taper implants in a mechanical fatigue test. Dent Res J (Isfahan).

[14] Mishra SK, Chowdhary R, Kumari S. Microleakage at the Different Implant Abutment Interface: A Systematic Review. J Clin Diagn Res. 2017;11(6):ZE10-ZE15. 10.7860/JCDR/2017/28951.10054 10.7860/JCDR/2017/28951.10054 PMC5535497 28764310

[15] Tallarico M, Fiorellini J, Nakajima Y, Omori Y, Takahisa I, Canullo L (2018). Mechanical outcomes, microleakage, and marginal accuracy at the implant-abutment interface of original versus nonoriginal implant abutments: a systematic review of* in vitro * studies. Biomed Res Int.

[16] Resende CC, Castro CG, Pereira LM (2015). Influence of the prosthetic index into morse taper implants on bacterial microleakage. Implant Dent.

[17] Sasada Y, Cochran DL (2017). Implant-Abutment Connections: A Review of Biologic Consequences and Peri-implantitis Implications. Int J Oral Maxillofac Implants.

[18] Perriard J, Wiskott WA, Mellal A, Scherrer SS, Botsis J, Belser UC (2002). Fatigue resistance of ITI implant-abutment connectors – a comparison of the standard cone with a novel internally keyed design. Clin Oral Implants Res.

[19] Liberati A, Altman DG, Tetzlaff J (2009). The PRISMA statement for reporting systematic reviews and meta-analyses of studies that evaluate health care interventions: explanation and elaboration. Ann Intern Med.

[20] Pollock D, Peters M, Khalil H (2023). Recommendations for the Extraction, Analysis, and Presentation of Results in Scoping Reviews. JBI Evid Synth..

[21] Sutter FHPW (1993). The new restorative concept of the Iti dental implant system: design and engineering. Int J Periodontics Restorative Dent.

[22] Yao KT, Kao HC, Cheng CK, Fang HW, Huang CH, Hsu ML (2015). The potential risk of conical implant-abutment connections: the antirotational ability of cowell implant system. Clin Implant Dent Relat Res.

[23] Semper W, Kraft S, Krüger T, Nelson K (2009). Theoretical considerations: implant positional index design. J Dent Res.

[24] Laleman I, Lambert F (2023). Implant connection and abutment selection as a predisposing and/or precipitating factor for peri-implant diseases: A review. Clin Implant Dent Relat Res.

[25] Soliman G, Guazzato M, Klineberg I, Chang MC, Ellakwa A (2021). Influence of Platform Switching, Abutment Design and Connection Protocols on the Stability of Peri-Implant Tissues. A Systematic Review. Eur J Prosthodont Restor Dent..

[26] da Silva-Neto JP, Nóbilo MA, Penatti MP, Simamoto PC Jr, das Neves FD. Influence of methodologic aspects on the results of implant-abutment interface microleakage tests: a critical review of in vitro studies. Int J Oral Maxillofac Implants. 2012;27(4):793–800. 22848880

[27] Sakka S, Baroudi K, Nassani MZ (2012). Factors associated with early and late failure of dental implants. J Investig Clin Dent.

[28] Zancopé K, Dias Resende CC, Castro CG, Salatti RC, Domingues das Neves F. Influence of the Prosthetic Index on Fracture Resistance of Morse Taper Dental Implants. Int J Oral Maxillofac Implants. 2017;32(6):1333–1337. 10.11607/jomi.4658 10.11607/jomi.4658 29140377

[29] Zhang WT, Cheng KJ, Liu YF (2022). Effect of the prosthetic index on stress distribution in Morse taper connection implant system and peri-implant bone: a 3D finite element analysis. BMC Oral Health.

[30] Hung HC, Huang CS, Pan YH (2019). The compressive strength of implant-abutment complex with different connection designs. J Dent Sci.

[31] Zancopé K, Resende CC, Tavares LN, Neves FD (2017). Influence of indexed abutments on the fracture resistance of internal conical dental implants. Gen Dent.

[32] Cerutti-Kopplin D, Rodrigues Neto DJ, Lins do Valle A, Pereira JR. Influence of reverse torque values in abutments with or without internal hexagon indexes. J Prosthet Dent. 2014;112(4):824–827. 10.1016/j.prosdent.2014.03.004 10.1016/j.prosdent.2014.03.004 24787130

[33] Hyun DG, Kwon HB, Lim YJ, Koak JY, Kim MJ. The Influence of a Positioning Hex on Abutment Rotation in Tapered Internal Implants: A 3D Finite Element Model Study. Int J Oral Maxillofac Implants. 2020;35(2):281–288. 10.11607/jomi.7673 10.11607/jomi.7673 32142564

[34] Michelon M, Milanos E, Lourenço EV, Telles DM. Do Oblique Cyclic Loads Influence the Tensile Strength of Different Morse Taper Connection Abutments?. Int J Oral Maxillofac Implants. 2019;34(5):1047–1052. 10.11607/jomi.7506 10.11607/jomi.7506 31528860

[35] Nokar S, Hajimiragha H, Sadighpour L, Mostafavi AS (2020). Evaluation of reverse torque values and failure loads of three different abutment designs with internal connections. Dent Res J (Isfahan).

[36] Yang F, Ruan Y, Liu Y (2022). Abutment mechanical complications of a Morse taper connection implant system: A 1- to 9-year retrospective study. Clin Implant Dent Relat Res.

[37] Gehrke SA, Scarano A, Cortellari GC, Fernandes GVO, Mesquita AMM, Bianchini MA (2023). Marginal bone level and biomechanical behavior of titanium-indexed abutment base of conical connection used for single ceramic crowns on morse-taper implant: a clinical retrospective study. J Funct Biomater.

[38] Szyszkowski A, Kozakiewicz M (2019). Effect of implant-abutment connection type on bone around dental implants in long-term observation: Internal cone versus internal hex. Implant Dent.

