# Supplementary material for: The mechanical and clinical influences of prosthetic index structure in Morse taper implant-abutment connection: a scoping review
Source: BMC Oral Health. 2023 Oct 21;23:775. doi: 10.1186/s12903-023-03545-3 (PMC10590505; doi:10.1186/s12903-023-03545-3)
Supplement: Supplementary file 1 — Additional file 1. [file 12903_2023_3545_MOESM1_ESM.docx]

**The** **mechanical and clinical influences of prosthetic index structure in Morse taper implant-abutment connection: a scoping review**

Liheng Shen^1^**^†^**, Chengzhi Dong^2^**^†^**, Jianping Chen^1^, Xiaolei Bai^3^, Fan Yang^1*^, Linhong Wang^1*^

^1^ Center for Plastic & Reconstructive Surgery, Department of Stomatology, Zhejiang

Provincial People's Hospital (Affiliated People's Hospital), Hangzhou Medical College, Hangzhou, Zhejiang, China.

^2^ Department of Stomatology, Zhejiang Chinese Medical University, Hangzhou, Zhejiang, China

^3^ Department of Stomatology, Zhejiang Provincial People's Hospital (Affiliated People's Hospital), Hangzhou Medical College, Hangzhou, Zhejiang, China

^†^ These authors contributed equally to this work and share first authorship

^*^ Correspondence: yangfan@hmc.edu.cn ; wanglinhong@hmc.edu.cn

Searching strategies

| **PubMed** | |
| --- | --- |
| #1 | "dental implant"[Mesh]" |
| #2 | ((((Morse Taper Dental Implant-Abutment Interface[Title/Abstract]) OR (Morse Taper Dental Implant Abutment Connection[Title/Abstract])) OR (Dental Implant Platform Switching[Title/Abstract])) OR (Dental Implant Abutment Designs[Title/Abstract])) OR (Morse taper[Title/Abstract]) |
| #3 | #1 OR #2 |
| #4 | "Dental Implant-Abutment Design"[Mesh] |
| #5 | ((((((internal hexagonal connection[Title/Abstract]) OR (external hexagonal connection[Title/Abstract])) OR (morse taper connection[Title/Abstract])) OR (prosthetic index[Title/Abstract])) OR (index[Title/Abstract])) OR (conical connection[Title/Abstract])) OR (hex[Title/Abstract]) |
| #6 | #4 OR #5 |
| #10 | #3 AND #6 |

| **Cochrane** | | |
| --- | --- | --- |
| #1 | | MeSH descriptor: [Dental Implant-Abutment Desige] explode all trees |
| #2 | | ((((((internal hexagonal connection OR (external hexagonal connection)) OR (morse taper connection) OR (prosthetic index)) OR (index)) OR (conical connection)) OR (hex) |
| #3 | | #1 AND #2 |
| **Web of Science** | | |
| #1 | (((((((TS=(Dental Implant-Abutment Design)) OR TS=(internal hexagonal connection)) OR TS=(external hexagonal connection)) OR TS=(morse taper connection)) OR TS=(prosthetic index)) OR TS=(index)) OR TS=(conical connection)) OR TS=(hex) | |
| #2 | (((((TS=(dental implant)) OR TS=(Morse Taper Dental Implant-Abutment Interface)) OR TS=(Morse Taper Dental Implant Abutment Connection)) OR TS=(Dental Implant Platform Switching)) OR TS=(Dental Implant Abutment Designs)) OR TS=(Morse taper) | |
| #3 | #1 AND #2 | |
| **Scopus** | | |
| #1 | INDEXTERMS(Dental Implant-Abutment Design) | |
| #2 | TITLE-ABS-KEY(internal hexagonal connection) OR TITLE-ABS-KEY(external hexagonal connection) OR TITLE-ABS-KEY(morse taper connection) OR TITLE-ABS-KEY(prosthetic index) OR TITLE-ABS-KEY(index) OR TITLE-ABS-KEY(hex) OR TITLE-ABS-KEY(conical connection) OR TITLE-ABS-KEY(dental implant) OR TITLE-ABS-KEY(Morse Taper Dental Implant-Abutment Interface) OR TITLE-ABS-KEY(Dental Implant Abutment Designs) | |
| #3 | #1 AND #2 | |
